# Reduced caterpillar damage can benefit plant bugs in Bt cotton

**DOI:** 10.1038/s41598-019-38917-9

**Published:** 2019-02-25

**Authors:** Michael Eisenring, Steven E. Naranjo, Sven Bacher, Angelique Abbott, Michael Meissle, Jörg Romeis

**Affiliations:** 1 Agroscope, Research Division Agroecology and Environment, Reckenholzstrasse 191, 8046 Zurich, Switzerland; 2 USDA-ARS, Arid-Land Agricultural Research Center, 21881N. Cardon Lane, Maricopa, AZ 85138 USA; 3 University of Fribourg, Fribourg, Department of Biology, Ecology & Evolution, Chemin du Musée 10, 1700 Fribourg, Switzerland

## Abstract

Bt cotton was genetically modified to produce insecticidal proteins targeting Lepidopteran pests and is therefore only minimally affected by caterpillar damage. This could lead to reduced levels of inherent, systemically inducible defensive compounds in Bt cotton which might benefit other important cotton herbivores such as plant bugs. We studied the effects of plant defense induction on the performance of the plant bug *Lygus hesperus* by caging nymphs on different food sources (bolls/squares) of Bt and non-Bt cotton which were either undamaged, damaged by Bt tolerant caterpillars, or treated with jasmonic acid (JA). Terpenoid induction patterns of JA-treated and *L*. *hesperus*-damaged plants were characterized for different plant structures and artificial diet assays using purified terpenoids (gossypol/heliocide H1/4) were conducted. Nymphs were negatively affected if kept on plants damaged by caterpillars or sprayed with JA. Performance of nymphs was increased if they fed on squares and by the Bt-trait which had a positive effect on boll quality as food. In general, JA-sprayed plants (but not *L*. *hesperus* infested plants) showed increased levels of terpenoids in the plant structures analyzed, which was especially pronounced in Bt cotton. Nymphs were not negatively affected by terpenoids in artificial diet assays indicating that other inducible cotton responses are responsible for the found negative effects on *L*. *hesperus*. Overall, genetically engineered plant defenses can benefit plant bugs by releasing them from plant-mediated indirect competition with lepidopterans which might contribute to increasing numbers of hemipterans in Bt cotton.

## Introduction

The cultivation of insect-resistant genetically engineered crops producing Cry proteins from *Bacillus thuringienis* (Bt crops) helps to control a range of key lepidopteran and coleopteran pest species while reducing the amount of chemical insecticide applications^1,2^. The area-wide use of Bt-transgenic crops, mainly maize (*Zea mays)* and cotton (*Gossypium hirsutum*), has led to significant population declines in target pests^3–6^. However, increasing numbers of herbivorous pests not targeted by the Cry proteins have been reported from Bt crops. This is the case in particular for Bt cotton where sucking bugs, such as plant bugs (Heteroptera: Miridae) and stink bugs (Heteroptera: Pentatomidae), have become problematic pests in some cropping systems^7–11^. Increased issues with non-target pests in maize, on the other hand, are mainly limited to the western bean cutworm (*Striacosta albicosta*) (Lepidoptera: Noctuidae)^12,13^. The reasons causing the increase in *S*. *albicosta* numbers are not fully understood but may include a variety of factors, such as reduced direct competition with herbivores targeted by the Bt trait, a reduction in insecticide use, as well as other ecological, agronomic and climatic causes^6^. In the case of Bt-transgenic cotton, several factors predisposing non-target pest to become more problematic have been suggested. Increases of sucking bugs in Bt cotton can mainly be attributed to a reduction in broad-spectrum insecticide applications as many insecticides against pest Lepidoptera also incidentally control other herbivore species^7,14^. In addition, there is mounting evidence that the strong reduction of lepidopteran populations in Bt cotton and, associated therewith, altered interspecific interactions among species also benefits non-target herbivores^15^. Stink bugs as well as cotton aphids, *Aphis gossypii* (Hemiptera: Aphididae) can benefit from the release of either direct interference competition or plant-mediated indirect competition with Bt-sensitive Lepidoptera^15–19^. There is evidence that plant-mediated indirect competition in cotton is partly driven by inducible defensive compounds. Best studied is a set of biosynthetically related non-volatile terpenoids (e.g. gossypol, heliocides, hemigossypolone) that are stored in subepidermal pigment glands^20,21^. These terpenoids are systemically induced in response to plant damage by tissue feeders^22–24^. They provide resistance primarily against lepidopterans, but may also be toxic to a range of other herbivores^21^. Reduced caterpillar damage on Bt cotton lowers the levels of inducible cotton defensive compounds, which in turn might improve the performance of non-target herbivores, as has been demonstrated for *A*. *gossypii*^17^. Plant-mediated indirect competition accounts for a major part of all interspecific herbivore interactions in natural ecosystems and can affect whole arthropod communities^25–27^. Thus, a release from indirect competition with caterpillars could also contribute to increasing numbers of sucking bugs in Bt cotton.

The western tarnished plant bug, *Lygus hesperus* (Hemiptera: Miridae) is a key herbivore in Bt cotton in the southwestern United States^11,28^. *L*. *hesperus* attacks mainly young cotton flower buds (squares), young bolls and growing points, where it feeds on enzymatically liquefied plant tissue^29,30^. This often leads to localized tissue necrosis and abortion of the attacked structure^29^. Literature documenting the role of cotton terpenoids as a defense mechanism against *L*. *hesperus* is limited. However, Tingey *et al*.^31^ and Ellington *et al*.^32^ showed that cotton varieties with low densities of gossypol-containing pigment glands positively affect *Lygus* spp. performance and population size.

Using *L*. *hesperus* as a model species we hypothesized that plant bugs benefit from reduced caterpillar damage in Bt cotton as they might profit from reduced levels of caterpillar-induced cotton defenses. This hypothesis was tested in the greenhouse, were we studied *L*. *hesperus* performance on Bt and non-Bt cotton subjected to different induction treatments. In further greenhouse and laboratory experiments we elucidated induction patterns of defensive cotton terpenoids in different plant structures that *L*. *hesperus* feeds on and studied their potential as explanatory factors affecting *L*. *hesperus* performance.

## Results

### Experiment 1: Effect of defense induction, food source and plant type on *L*. *hesperus* performance

**Figure 1:**
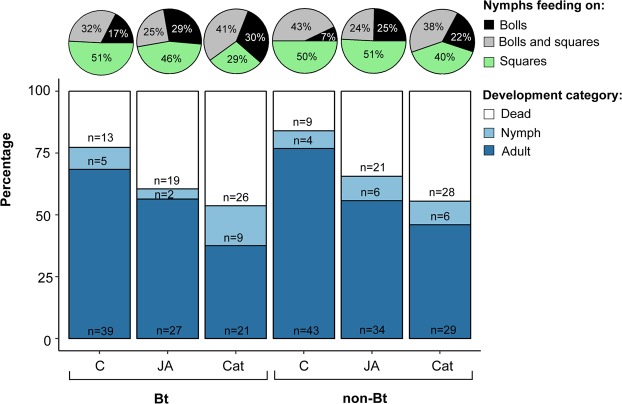
Percentage of *Lygus hesperus* nymphs that were kept on cotton (*Gossypium hirsutum*) for a maximum of 14 days on bolls, squares or both and either successfully developed into adults, remained in a nymphal stage or died. Bt and non-Bt cotton plants were either sprayed with jasmonic acid (JA), damaged by *Spodoptera exigua* caterpillars (Cat), or remained uninduced (C).

**Figure 2:**
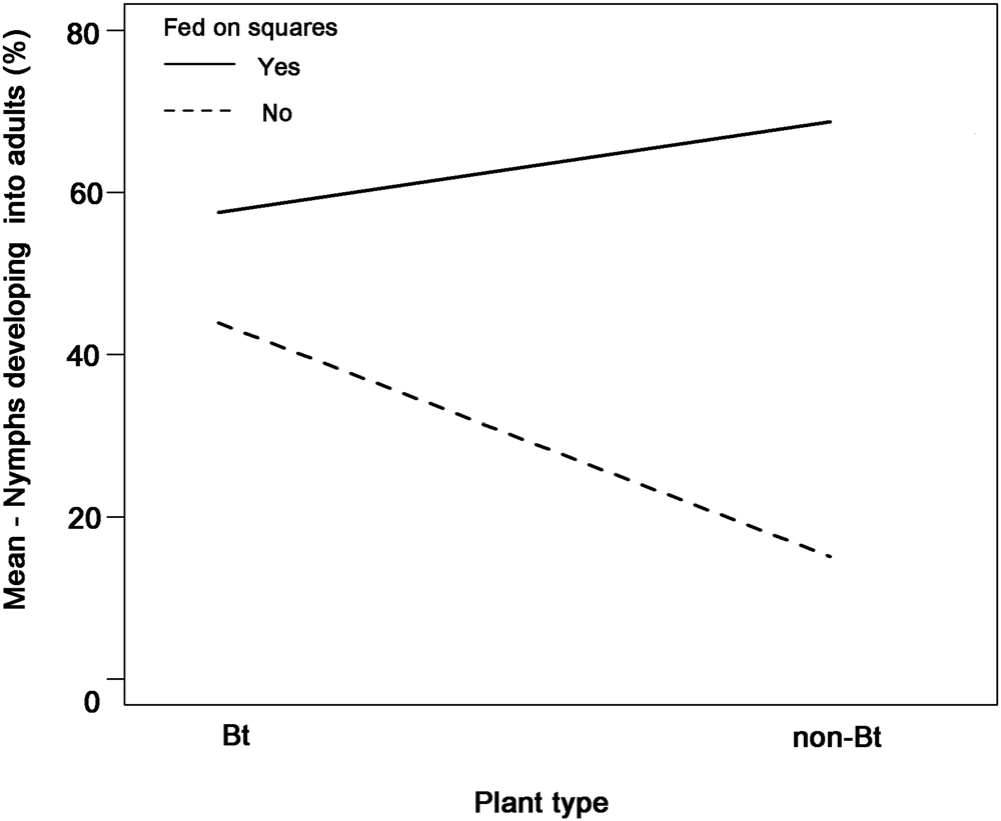
Percentage of *Lygus hesperus* nymphs (n = 341) that successfully developed into adults during the experiment. Nymphs were kept on Bt (n = 161) or non-Bt (n = 180) cotton (*Gossypium hirsutum*) and either fed on squares during their development (solid line) or exclusively fed on bolls (dashed line).

On average, mortality of *L*. *hesperus* on Bt and non-Bt plants was 25% and 20% higher when nymphs were kept on caterpillar- and JA-induced plants, respectively, in comparison to control plants (Fig. 1). Likewise, 30% and 10% fewer nymphs developed into adults on caterpillar- and JA-induced plants compared to controls, respectively (Fig. 1). The number of nymphs that died during the experiment and the number of nymphs that developed to adults (development rate) (Table 1) was significantly reduced on plants damaged by *Spodoptera* *exigua* (Lepidoptera: Noctuidae) caterpillars when compared to undamaged control plants. Similarly, plants sprayed with JA had a significant negative impact on nymph survival whereas the development rate was reduced, albeit not significantly, by JA (Table 1). Mortality and development rate was not only affected by damage treatments but was strongly dependent on food sources (Fig. 1). Feeding on squares had a strong positive effect on survival and development rate (Table 1). On average, plant type (Bt-trait) had no significant effect on mortality but a significant positive effect on the development rate (Table 1). A strong interaction between food source and plant type indicated that the detected effect of the Bt-trait could be mainly attributed to differences in boll qualities between the two plant types (Fig. 2, Table 1). More nymphs developed into adults when they exclusively fed on bolls of Bt-cotton when compared to nymphs only kept on bolls of non-Bt cotton (Fig. 2).

**Table 1:** Parameter estimates of the averaged candidate models explaining factors affecting survival or successful development of 3^rd^ instar *Lygus hesperus* nymphs into adults.

Survival				
Parameters	Estimate	SE	z value	p value
**Induction treatment**:				
Caterpillar	−1.03	0.37	2.79	0.005
JA	−0.71	0.35	2.01	0.04
**Food source:** Square	1.29	0.32	4.10	<0.001
Variables with no relevant explanatory power: Plant type, terpenoids, C:N ratio				
**Development rate**				
**Parameters**	**Estimate**	**SE**	**z value**	**p value**
**Induction treatment:**				
Caterpillar	−1.31	0.42	3.11	0.002
JA	−0.68	0.41	1.66	0.10
**Plant type:** Bt	1.54	0.70	2.20	0.03
**Food source:** Square	2.75	0.71	4.89	<0.001
**Interactions:** Square × Bt	−2.13	0.76	2.78	0.005
Variables with no relevant explanatory power: Terpenoids, C:N ratio				

Nymphs were kept for 14 days on squares, bolls or both of Bt or non-Bt-cotton plants that had been induced by *Spodoptera exigua* caterpillars, treated with jasmonic acid (JA), or remained uninduced.

In subsequent analyses, neither terpenoid concentrations nor C:N ratios measured in youngest leaves of experimental plants were significantly correlated with *L*. *hesperus* mortality or development success (all variables p > 0.05).

Weight gain of adult *L*. *hesperus* (max. 24 h old) was significantly negatively affected by bolls as a food source when compared with squares (estimate = −0.49 ± 0.18, z = 2.73, p = 0.006) whereas plant type (Bt-trait) and induction treatments had no significant effect on *L*. *hesperus* weight (all variables p > 0.05).

### Experiment 2: Cotton defense induction in fruiting structures and leaves

**Figure 3:**
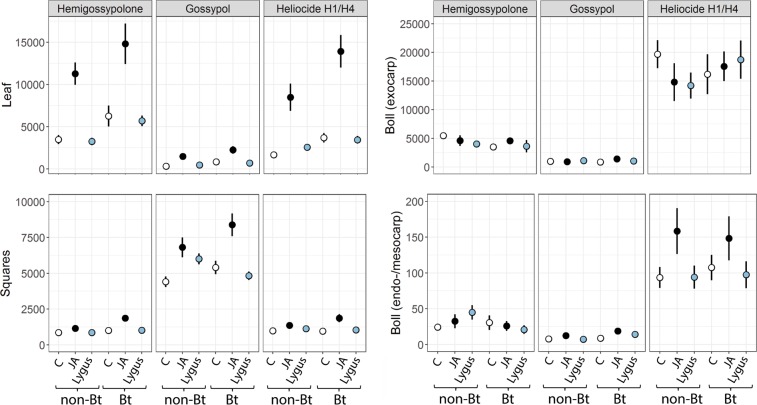
Mean (±SE) concentrations of terpenoids (ng/mg dw) in different plant structures of *Gossypium hirsutum*. Bt and non-Bt plants were either exposed to third instar *Lygus hesperus* (Lygus), treated with jasmonic acid (JA), or remained untreated (C). Seven days after treatment application, terpenoid concentrations were measured in bolls, squares and the youngest leaves. Note the different scale of the y-axes.

Terpenoid production in the youngest leaves, and to a lesser degree, also in squares, and the boll endo-and mesocarp was significantly affected by induction treatment and plant type (Table 2, Fig. 3). Post-hoc tests (not shown) revealed that the induction treatment effects can be attributed to JA. In leaves and squares, JA-sprayed plants had significantly higher terpenoid levels than controls or *L*. *hesperus-*damaged plants. In the boll endo- and mesocarp, terpenoid levels of JA-sprayed plants were significantly higher than levels of *L*. *hesperus*-damaged plants whereas control plants showed intermediate levels. Furthermore, Bt plants showed on average higher terpenoid levels in leaves, squares and the boll endo- and mesocarp than non-Bt plants (Table 2). The boll exocarp showed no signs of induction by any treatment. Levels of different terpenoids varied strongly among different plant structures (Fig. 3). JA-induced levels of gossypol were about 4 times higher in squares when compared to leaves, 5 times higher than in the boll exocarp and 500 times higher when compared to boll endo-and mesocarp. In contrast, induced levels of hemigossypolone in young leaves were 7–10 times higher when compared to square levels and 3–550 times higher than in boll structures. JA-induced as well as constitutive heliocide H1/H4 levels were highest in the boll exocarp followed by the JA-induced levels measured in leaves. Heliocide H1/H4 levels measured in squares and boll meso/endocarp were 1–2 orders of magnitude lower compared with levels measured in leaves or the exocarp.

**Table 2:** Impact of infestation treatments, plant type and the interactions of both variables on terpenoid concentrations in different cotton plant structures.

		Df	Hemigossypolone		Gossypol		Helicoide H1/H4	
			F	p	F	p	F	p
Boll (exocarp)	Plant type	1,67	2.95	0.09	0.40	0.53	0.29	0.59
	Induction treatment	2,67	1.53	0.22	0.81	0.45	0.39	0.68
	Plant: Treatment	2,67	1.25	0.29	3.50	**0**.**04**	1.46	0.24
Boll (endo-/mesocarp)	Plant type	1,67	1.94	0.17	9.24	**<0**.**01**	0.29	0.60
	Induction treatment	2,67	0.31	0.73	5.31	**<0**.**01**	3.64	**0**.**03**
	Plant: Treatment	2,67	1.60	0.21	2.43	0.1	0.18	0.84
Square	Plant type	1,68	9.10	**<0**.**01**	1.86	0.18	0.81	0.37
	Induction treatment	2,68	12.02	**<0**.**01**	15.15	**<0**.**01**	7.7	**<0**.**01**
	Plant: Treatment	2,68	1.66	0.20	4.03	**0**.**02**	1.08	0.35
Leaf	Plant type	1,69	12.11	**<0**.**01**	14.49	**<0**.**01**	16.13	**<0**.**01**
	Induction treatment	2,69	27.36	**<0**.**01**	19.51	**<0**.**01**	35.41	**<0**.**01**
	Plant: Treatment	2,69	0.19	0.83	0.60	0.55	1.04	0.36

Significant p values (p < 0.05) are shown in bold. Plotted means (±SE) of terpenoids are shown in Fig. 3.

### Experiment 3: Effect of terpenoids on *L*. *hesperus* performance

Different diet treatments had no significant effect on the total number of days that *L*. *hesperus* nymphs needed to develop into adults (7.97 ± 0.07 days) or the number of days they spent in the third, fourth, or fifth instar (Kruskal-Wallis; df = 3, all p > 0.18). Likewise, neither survival (Chi-square = 2.18, df = 3, p = 0.54), nor weight gain (fw = 7.68 ± 0.15 mg) was affected by different diets (ANOVA; F_3, 62_ = 0.137, p = 0.94).

## Discussion

The area-wide use of Bt-crops has significantly affected species composition in agro-ecosystems. In Bt-cotton, hemipterans such as plant and stink bugs have in many cases increased in relevance as pests^7–11^. While this is mainly due to the fact that the use of broad-spectrum insecticides is reduced in Bt cotton, our study indicates that a release from plant-mediated indirect competition with caterpillars could have additionally contributed to the increasing numbers of plant bugs in some Bt cotton systems.

### Impact of cotton defense induction and cotton terpenoids on *L*. *hesperus* performance

We found that cotton defense induction by *S*. *exigua* caterpillars had a negative effect on *L*. *hesperus* survival and development rate (but not weight gain) (Table 1). Similarly, plants sprayed with JA negatively affected *L*. *hesperus* performance (Table 1). This can most likely be attributed to JA-mediated cotton defense induction. However, we cannot rule out that also EtOH, which was used to dissolve JA, negatively affected *L*.*hesperus* performance since we did not control for any potential negative effects of EtOH. However, the low concentration of EtOH applied to the plants (100 µl/60 ml), the high volatility of EtOH as well as the finding that artificial diet containing EtOH did not negatively affect *L*.* hesperus* nymphs (experiment 3) speak against a negative impact of EtOH on *L*.*hesperus* in our experiment.

Plant-mediated interactions among herbivores, which are linked to alterations in plant defense levels, are well documented for a range of plant-herbivore systems^33–35^. In agreement with our results, previous studies reported that caterpillar-damaged cotton can adversely affect hemipteran species, i.e. cotton aphids, *A. gosypii* and the stink bugs, *Nezara viridula* and *Euschistus servus* (both: Hemiptera: Pentatomidae)^15,36^. There is evidence to suggest that these effects are caused by the induction of defensive terpenoids as a response to caterpillar damage or treatment with JA^23,36^. This is also supported by studies documenting that *Lygus* spp. have higher survival rates and occur in higher densities on glandless cotton varieties with comparatively low levels of terpenoids^31,32^.

However, when we exposed *L*. *hesperus* nymphs to pure gossypol or heliocide H1/H4 mixed into artificial diet, we observed no negative effects on their performance. In the case of heliocides H1/H4, the results need to be interpreted with caution since the purity of the compound used in our study was only ca. 50% and the concentration was about two orders of magnitude lower than concentrations found in squares or leaves of induced cotton plants. We can thus not rule out that heliocide H1/H4 in higher concentrations and purity might affect *L*. *hesperus*. In contrast, the purity of the gossypol used was high and concentrations in the artificial diet study were on the upper limit of concentrations measured in induced cotton leaves. Gossypol is known to be light-sensitive^37^. In order to minimize gossypol degradation in our study, terpenoid containing diet packs were exchanged every 4–5 days. Other artificial diet studies using similar concentrations of gossypol, showed that it remains bioactive during this period of time^38,39^. That gossypol has no adverse effect on *L*. *hesperus* is also supported by the results of our greenhouse experiment. Squares as a food source had a positive effect on *L*. *hesperus* performance when compared with bolls despite the fact that squares contained much higher concentrations of gossypol (Fig. 3). That cotton terpenoids might not be responsible for plant-mediated indirect competition between caterpillars and sucking bugs was also suggested by Zeilinger *et al*.^19^. They found that the boll-feeding stink bug *E*. *servus* avoided cotton plants damaged by caterpillars of *Helicoverpa zea* (Boddie) (Lepidoptera: Noctuidae) while it was attracted to plants damaged by *Heliothis virescens* Fabricius (Lepidoptera: Noctuidae). This, despite the fact that the concentration of terpenoids was significantly greater in seeds of *H*. *virescens*-damaged plants when compared to *H*. *zea*-damaged plants.

Given the large array of different cotton defenses against caterpillars, it is most likely that other potentially inducible defense mechanisms, such as chlorogenic acid, condensed tannins, or other phenolic compounds might explain the negative effects of cotton induction on *L*. *hesperus* performance^19,21,40^. Although C:N ratios in plants had no effect on *L*. *hesperus* performance, we cannot rule out that other changes in cottons nutritional quality affected *L*. *hesperus* as it has been reported that caterpillar damage can affect amino acid composition, water content or the oxidative status in cotton^40,41^.

### Cotton terpenoid induction in different plant structures

Terpenoid production in the boll exocarp was not inducible by any treatment, but JA-sprayed plants showed higher terpenoid levels in leaves and squares and to a lesser degree also in the boll endo/mesocarp. In contrast, *L*. *hesperus* feeding led to no terpenoid induction in any of the plant structures, indicating that nymphs did not trigger the induction of terpenoids in cotton. While chewing herbivores generally induce JA-related defenses it has been found that many sucking hemipterans like aphids or whiteflies can bypass such plant defense responses^42,43^. However, to what degree *L*. *hesperus* can manipulate the array of other cotton defenses is not well understood. Studies by Rodriguez-Saona *et al*.^44^ and Williams *et al*.^45^ show that *L*. *hesperus* feeding can induce volatiles comparable to caterpillar-induced volatile blends which might allow the plant to respond to *L*. *hesperus* infestations by, for example, attracting natural enemies.

### Impact of food source and plant type on *L*. *hesperus* performance

*L*. *hesperus* mortality and development, as well as weight gain, was strongly affected by food source (Table 1, Fig. 1). Feeding on squares had a positive effect on *L*. *hesperus* development and survival compared with individuals that had only access to bolls. Furthermore, adults that developed only on bolls where significantly lighter than adults that had fed on squares. Likewise, Chen and Parajulee^46^ showed that *L*. *hesperus* development duration and mortality was higher on cotton bolls compared to squares. It has been suggested that this might be explained by differences in nutritional quality of the two development stages of the reproductive structures^47^.

The Bt trait had a weak but significant positive effect on the number of nymphs that completed development. While the performance of nymphs that had access to squares during their development was only minimally affected by the Bt-trait, nymphs had a higher development success on bolls of Bt cotton in comparison to nymphs feeding on non-Bt cotton bolls (Fig. 2). Since concentrations of Cry proteins in squares and bolls of Bollgard II Roundup Ready Flex cotton are in a similar range^48^, this effect might not be due to direct (positive) effects of Cry-proteins on *L*. *hesperus* but can be attributed to other unknown changes in boll physiology present in the Bt plant.

In our greenhouse study, nymphs were not able to freely choose between bolls and squares. In the field, however, nymphs are less restricted in their food choice and likely prefer to feed on squares over bolls (Table 1, Fig. 2). Therefore, when compared with our results from the greenhouse, the food source parameter is probably less relevant in affecting *L*. *hesperus* performance under field conditions.

Herbivory can have profound effects on a host plants’ inducible responses which may entail a higher plant resistance to subsequent attacks by conspecifics or other herbivores. We demonstrated that the absence of such plant-mediated competition between herbivores can be an important additional factor contributing to the increased populations of plant bugs in Bt cotton in some regions. While the observed effects seem to be related to one of the many inducible mechanisms in cotton, it appears that the defensive terpenoid gossypol does not play a role in the cotton-*L*. *hesperus* interaction. Besides plant induction, *L*. *hesperus* performance was also affected by the Bt-trait and the food source. The latter might, however, be less relevant in the field where plant bugs can freely choose between different plant structures as food sources.

## Materials and Methods

### Plants and Insects

Commercial cotton plants (*G*. *hirsutum*), i.e., Bt cotton (Bollgard II Roundup Ready Flex cotton, DP1359B2RF, event MON15985 × MON1445, Monsanto, St. Louis, USA) and the genetically closest non-Bt cotton cultivar (Sure grow 125, Monsanto, St. Louis, USA) were individually grown under greenhouse conditions (25 ± 4 °C, av. 30% RH) in 3.8 l plastic pots containing a soil-sand mixture (9:5). Plants were watered daily and fertilized weekly using 100 ml of a 20% N, 20% P_2_O_5_, 20% K_2_O at 1 gl^−1^(=200 PPM N) Nutriculture General Purpose soluble fertilizer solution (Plant Marvel Laboratories, Chicago, USA). For all experiments 7–8 week old plants were used that possessed squares (flower buds) and young bolls (approx. 1 cm diameter). *L*. *hesperus* was reared under laboratory conditions (av. 27 °C, 30% RH, 14:10 L:D cycle). The first two instars were reared on green beans. Later instars and adults were kept on green beans plus an artificial diet described by Debolt^49^. For all experiments, only third instars with an initial weight of 0.5–0.9 mg were used. The colony was founded from collections in alfalfa and cotton fields in Maricopa, AZ USA in 2013. Bt-tolerant, fourth instar *S. exigua* were obtained from Frontier Agricultural Sciences (http://www.insectrearing.com).

### Experiment 1: Effect of cotton defense induction on *L. hesperus* performance

The aim of this experiment was to study the effect of cotton defense induction on *L*. *hesperus* performance. Bt and non-Bt cotton plants were exposed to one of the following induction treatments in the greenhouse: (i) plants were exposed to fourth instar *S*. *exigua*. A total of six larvae per plant were individually caged on single leaves equally distributed among top, medium and lower node regions using organdy cloth bags with Velcro that fastened around the petiole. Larvae were transferred to new leaves every 2–3 days. After 1 week all larvae and bags were removed; (ii) plants were induced with the plant hormone jasmonic acid (JA) (Sigma-Aldrich, St. Louis, USA), which is known to induce cotton defense responses^50^. Each plant was sprayed with a solution containing 60 ml distilled water and 5 mg of JA dissolved in 100 µl EtOH using a vaporizer^24^; (iii) plants remained untreated (control). This resulted in a total of six treatments (three induction treatments each for Bt and non-Bt plants). A total of 20–24 plants were subjected to each treatment. One week after application of the induction treatments, three freshly molted and weighed third instar *L*. *hesperus* nymphs were caged in individual organdy cloth bags with a Velcro fastener on each plant (experimental unit). Nymphs were randomly caged on branchlets with either a single young boll or a square as food source. Preliminary feeding studies using artificial diet^49^ showed that most third instar nymphs successfully developed into adults within 7–8 days (not shown). To make sure that the nymphs had enough time to develop into adults under greenhouse conditions they were kept on plants for a maximum of 14 days. After an initial period of five days, nymphs were checked daily during the experiment. Individuals that developed into adults were removed from plants and weighed again to calculate the weight gain.

*L*. *hesperus* nymphs were transferred to new squares or young bolls of the same plant if the plants aborted these structures due to *L*. *hesperus* feeding damage or if young bolls or squares developed into older bolls or flowers. Therefore, nymphs fed either only on squares, only on bolls or on both structures during the experiment. Mortality and the number of nymphs that survived but did not manage to develop into adults was recorded. The youngest leaf of each plant in all treatments was sampled and stored at -80 °C for further biochemical analyses (see measured terpenoids and plant nutrients) at the end of the experiment.

### Experiment 2: Cotton defense induction in different plant structures

To study to what degree JA and *L*. *hesperus* feeding induces defense responses in different cotton plant structures and if these induction patterns are in agreement with the results from experiment 1, Bt and non-Bt cotton plants were exposed to one of the following induction treatments in the greenhouse: (i) three freshly molted third instar *L*. *hesperus* nymphs were randomly caged in organdy cloth bags with a Velcro fastener on young bolls or squares of each plant for 7 days. Nymphs were transferred to new squares or bolls of the same plant if cotton plants dropped these structures or if young bolls or squares developed into old bolls or flowers. Infested plants where more than one nymph died during the experiment were discarded; (ii) plants were induced with JA (positive control, described above); (iii) plants remained untreated (negative control). This resulted in a total of six treatments (three induction treatments each for Bt and non-Bt plants). A total of 10–14 individual plants were subjected to each treatment. After 7 days, all nymphs were removed and a young, not yet fully expanded leaf from the top of each plant was collected. A random square and both the exocarp and the locule (endo-and mesocarp) of a random young boll that was not previously infested (approx. 1 cm diameter) were sampled if available on the plant, and stored at −80 °C for further biochemical analyses.

### Experiment 3: Effect of terpenoids on *L*. *hesperus* performance

An artificial diet study was conducted with freshly molted third instar nymphs to assess the sensitivity of *L*. *hesperus* to two terpenoids, gossypol and heliocide H1/H4. Nymphs were fed with artificial diet described by Debolt^49^, which was spiked with either: (i) 2 mg gossypol dissolved in 4.5 µl EtOH (95%)/g diet; (ii) artificial diet containing 0.9 mg heliocides H1/H4 dissolved in 4.5 µl EtOH (95%)/g diet; (iii) artificial diet containing just the EtOH solvent (4.5 µl EtOH (95%)/g diet); (iv) pure artificial diet (negative control); (iv) artificial diet containing the insecticide acephate (100 µl of a 29.4 mg/ml H_2_O solvent/g diet) (positive control). Gossypol (purity ≥95%) was purchased from Sigma-Aldrich (St. Louis, USA) heliocide H1/H4 (purity >50%) was extracted from young cotton leaves by C. Bochet at the University of Fribourg, Switzerland, according to Stipanovic *et al*.^51^, and acephate was purchased from AMVAC Chemical Company (Los Angeles, USA).

For each treatment, 18 *L*. *hesperus* nymphs were individually weighted and separately placed in ventilated Petri dishes (5.5 cm diameter) each containing a Parafilm pack filled with 1 g artificial diet. Diet packs were replaced every 4–5 days. For each nymph, survival and time spent in each instar was recorded for 2 weeks. After two weeks, when all surviving nymphs had developed into adults, individuals were weighed again and the weight gain was calculated. All treatment dishes were kept in an incubator (26 ± 0.4 °C, av. 40% RH, 14:10 L:D cycle).

### Chemical analyses of terpenoids and leaf nutrients

All collected plant parts were lyophilized and whole leaves, squares, a random piece of the boll exocarp and half of a boll locule were pulverized (30 Hz, 30 sec) in individual 2 ml Eppendorf tubes each containing a 3 mm tungsten carbide bead using a TissueLyser 2 mixer mill (Qiagen, Hilden, Germany). From each pulverized plant structure, 9-11 mg of powder was put in a 2 ml Eppendorf tube and extracted following the method of Benson *et al*.^52^. Briefly, 1 ml of a mixture of acetonitrile (≥99.9%, Scharlau, Barcelona, Spain), MilliQ-water and ortho-phosphoric acid (≥85%, Sigma-Aldrich) (80:20:0.1) was added to each Eppendorf tube. Tubes were then vortexed, ultrasonicated for 3 min and centifuged for 3 min (8 × *g*). The extracts were transferred to glass vials for analysis with a high-performance liquid chromatography (HPLC) system (1260 Infinity, Agilent Technologies, Santa Clara, USA, Varian Polaris Amide C-18 column, 150 × 2.0 mm, 3 µm, equipped with a precolumn C18, 4 × 3.0 mm, Supeloc Security Guard System). HPLC analyses followed the methodology described by Hagenbucher *et al*.^17^. Gossypol was identified by comparing the extract retention time with the retention time of a standard gossypol solution (gossypol from cotton seeds, ≥95% purity, Sigma-Aldrich). The retention times of the terpenoids hemigossypolone and heliocides H1/H4 were identified based on previously published chromatograms^52,53^. The identity of the terpenoids was furthermore confirmed by mass spectrometry. We were unable to confirm the identity of a distinct peak assigned to heliocide H2/H3 with massspectrometrical analyses. We therefore did not include heliocide H2/H3 in this study. Terpenoid concentrations were quantified in terms of gossypol equivalents^54^.

To quantify C:N ratios from leaves of greenhouse experiment 1, 4–5 mg of lyophilized tissue from each leaf was individually filled in tin capsules and the C:N ratios for each sample was subsequently measured by elemental analysis (Hekatech Euro EA 3000,Wegberg Germany) as described in Leifeld *et al*.^55^.

### Statistical analyses

For all statistical analyses, the Software R (version 3.2.3) was used^56^. The standard error of the mean is provided for all mean values (mean ± SE).

### Experiment 1: Effect of cotton defense induction on *L*. *hesperus* performance

The two response variables, nymphs that died within 14 days (binomial distribution) and nymphs that developed within 14 days into adults (binomial distribution), were analyzed using generalized linear mixed models (GLMM) (“glmer” function of the R-package lme4, version 1.1–12)^57^ with induction treatments (caterpillar damage, JA treated, and control), plant type (Bt and non-Bt cotton) and food source (squares and bolls) as explanatory variables. In a second step, the same response variables were analyzed using a GLMM with terpenoids (gossypol, heliocide H1/H4, hemigossypolone) and C:N ratios in the youngest leaves of the experimental plants as explanatory variables.

The response variable weight gain of individuals that developed into adults was analyzed using a linear mixed model (LMM) (“lmer” function of the lme4 package, version 1.1–12)^57^ with induction treatment and plant type (see above) as explanatory variables. In all models individual plants were used as random effects.

Factors affecting *L*. *hesperus* performance were identified with an information theoretic framework of model selection^58^. For each response variable, models were fitted with all possible combinations of the explanatory variables as well as their 2-way interactions using the “dredge” function of the R-package MuMIn (version 1.15.6)^59^. Models were then ranked according to Akaikes information criterion corrected for finite sample sizes (AICc). To determine the explanatory variables that best explained variation in *L*. *hesperus* performance, all models were selected that conformed to two rules: First, only models with a Δ AICc value of ≤6 were selected, i.e., all models whose AICc value was at most 6 higher than the lowest AICc obtained. Second, a model was only selected if its AICc value was less than the AICc value of all the simpler models within which it is nested, in order to avoid selecting overly complex models^60^. Finally, the values of all model parameters were estimated by model averaging among the set of candidate models chosen by the model selection procedure (function “model. avg” from the MuMIn package, version 1.15.6)^59^. This method weights parameter estimates of more credible models (i.e, with lower AICc) higher than those with lower credibility. Parameters that were not part of any model chosen by model selection can be considered as having no relevant explanatory power.

### Experiment 2: Cotton defense induction in different plant structures

The effect of the explanatory variables plant type (Bt and non-Bt cotton), induction treatment (*L*. *hesperus* infested, JA treated, and control) and their interaction on terpenoid concentrations in youngest leaves, squares, and developing bolls were analyzed using separate two-way ANOVAs using the “lm” function. In the case of significant effects of plant type or induction treatment, Tukey HSD post-hoc tests were conducted (package agricolae, version 1.2–4)^61^. Data were square root transformed prior to analysis to meet the assumptions for normality and homoscedasticity.

### Experiment 3: Effect of terpenoids on *L*. *hesperus* performance

Kruskal-Wallis tests followed by Holm-Bonferroni post-hoc tests (package agricolae) were used to test the effect of different diet treatments on total nymphal development time. Furthermore, the effect of the treatment on development times for each instar was tested separately. The effect of the treatment on net weight gain was analyzed using ANOVA followed by Tukey HSD post-hoc tests. Survival among different diets was compared using a Chi-square test (package gmodels version 2.16.2)^62^. Nymphs fed with acephate spiked diet (positive control) died within 24 h after the experiment started and were therefore not included in the analyses.
